# Effect of Nutritional Intervention Programs on Nutritional Status and Readmission Rate in Malnourished Older Adults with Pneumonia: A Randomized Control Trial

**DOI:** 10.3390/ijerph16234758

**Published:** 2019-11-27

**Authors:** Pei-Hsin Yang, Meng-Chih Lin, Yi-Ying Liu, Chia-Lun Lee, Nai-Jen Chang

**Affiliations:** 1 Department of Sports Medicine, Kaohsiung Medical University, Kaohsiung 807, Taiwan; wendy0962@cgmh.org.tw; 2 Department of Nutritional Therapy, Kaohsiung Chang Gung Memorial Hospital, Kaohsiung 833, Taiwan; 3 Division of Pulmonary and Critical Care Medicine, Department of Internal Medicine, Kaohsiung Chang Gung Memorial Hospital, Chang Gung University College of Medicine, Kaohsiung 833, Taiwan; mengchih@cgmh.org.tw; 4 Department of Nursing, Kaohsiung Chang Gung Memorial Hospital, Kaohsiung 833, Taiwan; nicole@cgmh.org.tw; 5 Center for Physical and Health Education, National Sun Yat-sen University, Kaohsiung 804, Taiwan; karenlee1129@gmail.com; 6 Ph.D. Program in Biomedical Engineering, Kaohsiung Medical University, Kaohsiung 807, Taiwan; 7 Regenerative Medicine and Cell Therapy Research Center, Kaohsiung Medical University, Kaohsiung 807, Taiwan

**Keywords:** nutritional intervention, malnutrition, hospital stay, family care, caregiver, respiratory disease

## Abstract

Pneumonia leads to changes in body composition and weakness due to the malnourished condition. In addition, patient family caregivers always have a lack of nutritional information, and they do not know how to manage patients’ nutritional intake during hospitalization and after discharge. Most intervention studies aim to provide nutritional support for older patients. However, whether long-term nutritional intervention by dietitians and caregivers from patients’ families exert clinical effects—particularly in malnourished pneumonia—on nutritional status and readmission rate at each interventional phase, from hospitalization to postdischarge, remains unclear. To investigate the effects of an individualized nutritional intervention program (iNIP) on nutritional status and readmission rate in older adults with pneumonia during hospitalization and three and six months after discharge. Eighty-two malnourished older adults with a primary diagnosis of pneumonia participated. Patients were randomly allocated to either a nutrition intervention (NI) group or a standard care (SC) group. Participants in the NI group received an iNIP according to energy and protein intake requirements in addition to dietary advice based on face-to-face interviews with their family caregivers during hospitalization. After discharge, phone calls were adopted for prescribing iNIPs. Anthropometry (i.e., body mass index, limb circumference, and subcutaneous fat thickness), blood parameters (i.e., albumin and total lymphocyte count), hospital stay, Mini-Nutritional Assessment-Short Form (MNA-SF) score, target daily calorie intake, total calorie intake adherence rate, and three-major-nutrient intakes were assessed during hospitalization and three and six months after discharge. Both groups received regular follow-up through phone calls. Furthermore, the rate of readmission resulting from pneumonia was recorded after discharge. During hospital stay, the NI group showed significant increases in daily calorie intake, total calorie intake adherence rate, and protein intake compared with the SC group (*p* < 0.05); however, no significant difference was found in anthropometry, blood biochemical values, MNA-SF scores, and hospital stay. At three and six months after discharge, the NI group showed significantly higher daily calorie intake and MNA-SF scores (8.2 vs. 6.5 scores at three months; 9.3 vs. 7.6 scores at six months) than did the SC group (*p* < 0.05). After adjusting for sex, the readmission rate for pneumonia significantly decreased by 77% in the NI group compared with that in the SC group (*p* = 0.03, OR: 0.228, 95% CI: 0.06–0.87). A six-month iNIP under dietitian and patient family nutritional support for malnourished older adults with pneumonia can significantly improve their nutritional status and reduce the readmission rate.

## 1. Introduction

According to the World Health Organization, 450 million people develop pneumonia each year, and approximately four million people die from this disease, accounting for 7% of the global population [1]. Pneumonia is defined as an infection process of the lung parenchyma, which results from the invasion and overgrowth of microorganisms, breaking down defenses, and provoking intra-alveolar exudates [2]. Signs and symptoms of pneumonia may include chest pain, cough, fatigue, fever, nausea, vomiting or diarrhea, and shortness of breath. In addition, in a less active lifestyle, the consequence of the patients with pneumonia leads to malnutrition and higher mortality rates [3]. Patients with pneumonia become malnourished (e.g., protein-calorie malnutrition), exhibit declining health and changes in weight loss, and seriously impair respiratory muscle contractility and endurance [4].

Malnutrition leads to the development of pneumonia and weakens the physical activity and immune system [5]. Therefore, adequate nutrition directly aids respiratory muscle function and immune defense mechanisms and provides high immunity against environmental pathogens in the lungs to reduce potential disease progression [6,7]. Therefore, the major role of nutrition in alleviating pneumonia is reducing malnutrition that induces high mortality and morbidity [8,9] and maintaining impaired respiratory muscle contractility [10]. Thus, nutritional intervention is vital in patients with pneumonia.

The goal of nutritional intervention is to decrease malnutrition, thereby reducing morbidity, delaying mortality, delaying disease progression, and improving respiratory function [11]. The Mini-Nutritional Assessment (MNA) score has been used to assess the nutritional status of older adults in nursing homes [12]. To date, most intervention studies have aimed to provide nutritional support for older patients to improve nutritional status [13], reduce hospitalization costs, and reduce the length of stay and the number of readmissions [14,15]. However, most of these studies have mainly recurred from older adults with chronic obstructive pulmonary disease (COPD) [15] or community-dwelling older adults [16], rather than older patients with pneumonia, which is a life-threatening disease, in particular. However, whether long-term nutritional intervention by dietitians and caregivers from patients’ families exert clinical effects—particularly in malnourished pneumonia—on nutritional status and readmission rate at each interventional phase from hospitalization to postdischarge remains unclear. Furthermore, patient family care, which is one of the environmental factors, influences the patient’s food and nutritional intake [17]. However, patients’ families always have a lack of nutritional information, and they do not know how to manage patients’ nutritional intake during hospitalization and particularly after discharge [18]. Consequently, it may place patients at higher risk of malnutrition. To combat malnutrition, continuous nutrition intervention should be accessible, sustainable, and integrated with health care providers (e.g., dietitian) [19]. Importantly, family caregivers are advised to understand the individualized nutrition information for patients that may prevent and improve patient under-nutrition [20]. Therefore, the aim study was to investigate the effects of an individualized nutritional intervention program when delivered through mutual care by a dietitian and patient family caregivers in older adults with pneumonia during hospitalization and three and six months after discharge. The primary outcome was nutritional status (i.e., MNA scores). The secondary outcomes were assessed using anthropometric measurements, blood biochemical values, daily calorie intake, hospital stay, and readmission rate.

## 2. Methods

### 2.1. Study Design and Setting

**Figure 1:**
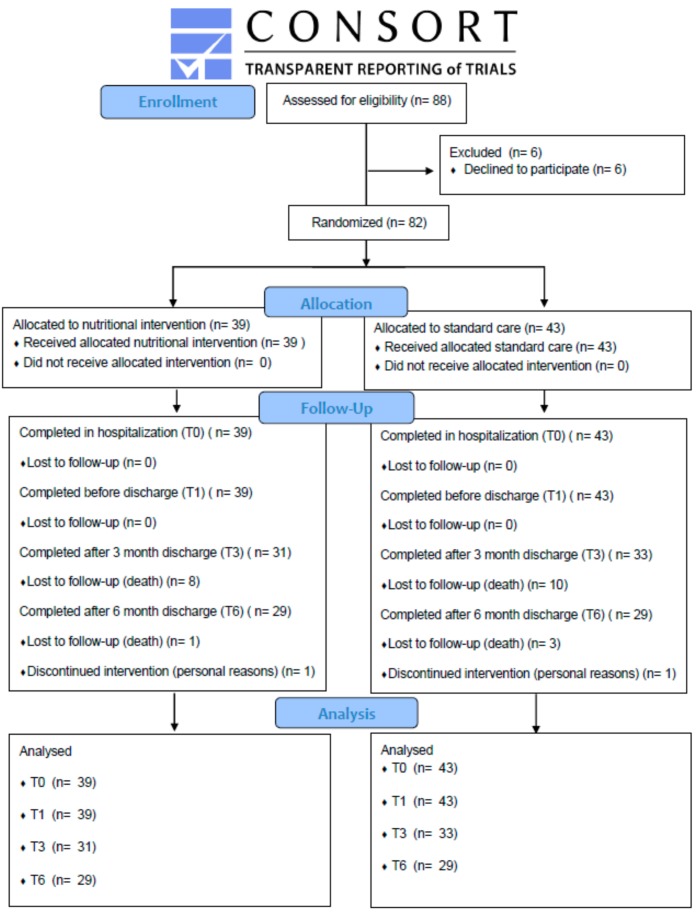
CONSORT flow diagram.

This study was approved by the Institutional Review Board of Chang Gung Medical Foundation (Approval No. 201700126B0C501), based on current legislation and performed in accordance with the Declaration of Helsinki [21]. This study protocol was registered with ClinicalTrials.gov (NCT04160819). This study was a prospective, single-center, randomized control trial. Regarding the recruitment process, we enrolled older malnourished adults with a primary diagnosis of pneumonia who were treated in Kaohsiung Chang Gung Memorial Hospital from March 2017 to May 2018 and received a nutrition support team from the Nutrition Department. Because of the concern of patients’ consciousness level, researchers explained the study purpose to their family caregivers and obtained their written informed consent before starting the study. Subsequently, an independent clinical staff member who was not involved in the recruitment prepared random allocation cards (A lot: NI group; B lot: SC group) in sealed, opaque envelopes. A researcher drew and opened the envelope and notified participants of the group assignment. However, it was difficult to blind the family caregivers to group assignment. A total of 82 eligible participants were randomly allocated to receive either nutrition intervention (NI) or standard care (SC) (Figure 1). Patients who received a primary diagnosis of pneumonia were identified initially from the Health Care Information System of Kaohsiung Chang Gung Memorial Hospital by a physician. The participants were the NI or SC group. At the 6 month follow-up, 58 of 82 patients with pneumonia completed this trial (Figure 1).

### 2.2. Study Participants

Inclusion criteria were as follows: primary diagnosis of pneumonia by a physician, age more than 65 years, and malnutrition status indicated by body mass index (BMI) <18.5 kg/m^2^ or MNA-Short Form (MNA-SF) score ≤7. Exclusion criteria were as follows: renal insufficiency (glomerular filtration rate [GFR] <60 mL/min/1.73 m^2^ or GFR staging of G3b–G5), cancer hospital stay <7 days.

### 2.3. Interventions

The NI group was provided support by a dietitian who elaborated an individualized nutritional plan for each participant based on their nutritional status and physical activity, taught the postdischarge diet, and provided dietary advice. Because of the concern of patients’ consciousness, their family caregivers participated in the dietary counseling, and they were taught by a dietitian. After discharge, phone calls were adopted for tracking the nutritional intake status and prescribing individualized nutritional plans. The SC group was only provided standard nutritional supplements according to the Kaohsiung Chang Gung Memorial Hospital Nutrition Department, and patients’ family caregivers were not provided dietary advice.

### 2.4. Outcomes Measures

Data collectors from clinical staffs were trained on data collection procedures and follow-up through phone calls. The dietitian was in charge of anthropometry, the MNA-SF score, and the nutritional intake status. In addition, the blood parameters were performed by the Department of Laboratory Medicine from Kaohsiung Chang Gung Memorial Hospital.

#### 2.4.1. Primary Outcomes

Mini-Nutritional Assessment -Short Form (MNA-SF) scores can be used to indicate the presence of malnutrition in older adults with diseases such as diabetes, pneumonia, and hypertension [22]. MNA-SF comprises simple measurements and short questions that can be completed in approximately 10 min. MNA-SF has high reliability, with an intraclass correlation coefficient (ICC) of 0.83, and has high sensitivity (97.9%) and specificity (100%) [23]. MNA-SF also predicts mortality and hospitalization costs. Most importantly, before a major change in body weight or albumin levels occurs, people at risk of malnutrition are more likely to reduce their caloric intake and can be provided nutritional intervention. MNA-SF scores ranging within 0–7, 8–11, and 12–14 indicate malnutrition, risk of malnutrition, and normal nutritional status, respectively [24].

#### 2.4.2. Secondary Outcomes

Anthropometric measurements, blood biochemical analysis, calorie needs, intake assessment, calorie intake adherence rate, hospital stay, and readmission rate were assessed. BMI was determined by dividing weight (kg) by height (m^2^). BMI was determined by dividing weight (kg) by height (m^2^). The rate of death from respiratory diseases and aging has been reported to increase in underweight (BMI < 18.5 kg/m^2^) groups [25]. Body circumference and subcutaneous fat thickness were measured by determining the upper arm circumference (AC), triceps skinfold (TSF), and arm muscle circumference (AMC) [26]. AMC and arm muscle area (AMA) were calculated as follows: AMC (mm) = AC (mm) − (π × TSF) and AMA (mm^2^) = (AC (mm) − (π × TSF)) × 2/4π [27].

All Blood biochemical analysis was performed by the Department of Laboratory Medicine from Kaohsiung Chang Gung Memorial Hospital. It comprised albumin (normal range, 3.5–5.0 g/dL), white blood cell (WBC, normal range, 3.9–10.6 × 10 ^3^ cells/µL in men and 3.5–11 × 10 ^3^ cells/µL in women), lymphocyte (normal range, 20%–56%), and total lymphocyte count (TLC; normal range, 2–3.5 × 10 ^3^ cells/mm^3^; mild malnutrition <1.8 × 10 ^3^ cells/mm^3^; severe malnutrition <0.8 × 10 ^3^ cells/mm^3^); the albumin samples were centrifuged at 3300 rpm (2280 × g) for 10 min (KUBOTA 8420 High Capacity Tabletop Centrifuge); complete blood count was performed on a Sysmex XE-5000 analyzer XN^®^ (Sysmex, Kobe, Japan). Data on these parameters were obtained from the electronic medical record system of the medical center.

The calorie needs of patients were estimated by the dietitian through phone calls. The dietitian continuously monitored changes in an individual patient’s body composition and blood biochemical values to immediately adjust calorie needs. Calorie (energy) needs were determined using the following formula: calorie needs = ideal body weight (IBW) × calories needed for activity. IBW was calculated using the following formula: IBW = height 2 (m^2^) × 22 BMI [28]. Regarding the intake assessment method, the 24 h dietary recall method is simple, consumes less time, and exerts lower patient burden, resulting in a higher participation rate. It can effectively and correctly provide the daily calorie intake data of patients [29]. However, responses requiring 24 h dietary recall are often not obtained from elderly people because of social desirability or memory problems. Therefore, in this study, the dietitian used 24 h dietary recall methods from family caregivers’ responses to determine the daily calorie intake data of patients during hospitalization and 3 and 6 months after discharge. The calorie intake adherence rate was determined using the following formula: calorie intake adherence rate = (actual daily calorie intake (kcal/day)/calorie needs (kcal/day)) × 100%.

### 2.5. Statistical Analysis

All analyses were conducted using the SPSS statistical package (version 22.0; SPSS, Inc., Chicago, IL, USA). The data were normally distributed by the Shapiro–Wilk test, and the homogeneity of variance was confirmed by the Levene’s test. For comparisons between the NI and SC groups, an for examining sex and comorbidities; and Fisher’s exact test for exploring the readmission diagnosis. The generalized estimating equation (GEE) was adopted to test the time × group interaction, main effect of time, and main effect of the group [30]. Sex was included as a significant covariate in analyses. Data are expressed as the mean ± standard deviation and as the number or percentage. A *p*-value of <0.05 indicates statistical significance. A sample size calculation based on anticipated differences in MNA-SF scores as the primary outcome was estimated based on an anticipated mean difference of 1.5 scores (standard deviation 2) between the groups at the end of postintervention. The calculation was based on an alpha level of 0.05 and a desired power of 80%. A minimum sample size of 30 patients per group was used. In addition, assuming a dropout rate of 30% [31], we enrolled at least 39 participants in each group. In this study, the post hoc power was 80.25%.

## 3. Results

### 3.1. Baseline Characteristics of Patients

No significant differences were observed in age, the readmission rate, and number of comorbidities (except for dementia and other lung diseases) between the groups, but significant differences were observed in sex (*p* < 0.044) (Table 1).

### 3.2. MNA-SF Assessment

**Figure 2:**
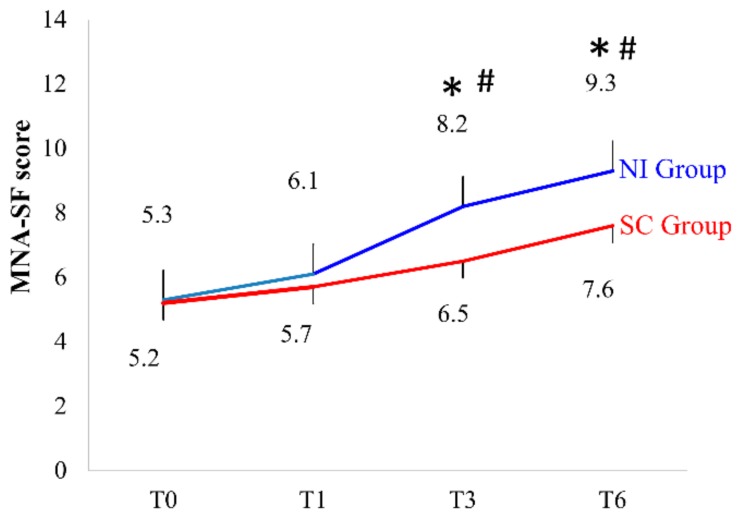
Mini-Nutritional Assessment-Short Form (MNA-SF) scores of the nutritional intervention group and the standard care group; * *p* < 0.05 indicates a significant difference between the groups. # *p* < 0.05 indicates a significant difference compared to T0. T0 = during hospitalization; T1 = before discharge; T3 = 3 months after discharge; T6 = 6 months after discharge. *n* = 39 for NI, 43 for SC at T0; *n* = 39 for NI, 43 for SC at T1; *n* = 31 for NI, 33 for SC at T3; *n* = 29 for both NI and SC at T6, respectively.

For the MNA-SF score, the time × group interaction was not significant (*p* = 0.05) (Figure 2). However, the main effect of time (*p* < 0.001) and the main effect of the group (p = 0.033) were significant. MNA-SF scores varied significantly with time (*p* < 0.001). Particularly, the NI group had significantly increased scores at three and six months after discharge (T3 and T6, respectively) compared with that at baseline (T0). Furthermore, the NI group had significantly higher scores than the SC group at T3 and T6.

### 3.3. Anthropometric Measurements

The time × group interaction and the main effect of the group were not significant for BMI, TSF, AC, AMC, and AMA. However, the main effect of time was significant for all anthropometric measurements (*p* = 0.01 for BMI; *p* = 0.023 for TSF; *p* < 0.001 for AC, AMC, and AMA) (Table 2).

### 3.4. Blood Analysis

The time × group interaction and the main effect of the group were not significant for albumin, WBC, lymphocyte, and TLC. However, the main effect of time was significant for WBC and lymphocyte count (both *p* < 0.001) (Table 3).

### 3.5. Assessment of Total Daily Calorie Intake, Calorie Intake Adherence Rate, and Three-Nutrient Ratios

**Figure 3:**
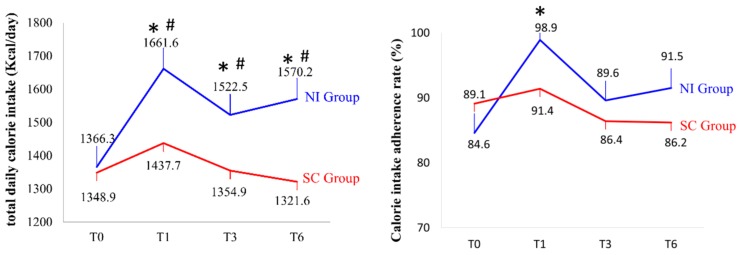
Daily intake and calorie intake adherence rate in the NI and SC groups. * *p* < 0.05 indicates a significant difference between the groups. # *p* < 0.05 indicates a significant difference compared to T0. *n* = 39 for NI, 43 for SC at T0; *n* = 39 for NI, 43 for SC at T1; *n* = 31 for NI, 33 for SC at T3; *n* = 29 for both NI and SC at T6, respectively.

For the total daily calorie intake (*p* = 0.05), calorie intake adherence rate (*p* = 0.092), and protein (*p* = 0.117), lipid (*p* = 0.232), and carbohydrate (*p* = 0.116) intake ratios, the time × group interaction was not significant (Figure 3, Table 4). However, the main effect of time was significant for the daily calorie intake, calorie intake adherence rate, and protein and lipid intake ratios (all *p* < 0.001). Furthermore, the main effect of time was significant for the daily calorie intake and protein and lipid intake ratios, indicating that the calorie intake of the NI group was higher than that of the SC group. The calorie intake adherence rate was 5.3% higher in the NI group than in the SC group (*p* = 0.003) before and after discharge. Furthermore, the calorie intake adherence rate of the NI group was higher than that of the SC group by 3.2% and 5.3% at three and six months after discharge, respectively. Moreover, protein intake was also significantly higher in the NI group than in the SC group before discharge and three and six months after discharge (*p* < 0.001, *p* = 0.023, *p* = 0.001, respectively). The lipid intake was also higher in the NI group than in the SC group before discharge, in the third month after discharge, and in the sixth month after discharge, with significant differences (all *p* < 0.001). However, no significant difference was observed in the calorie intake adherence rate and carbohydrate intake ratio.

### 3.6. Hospital Stay and Readmission Rate

No significant difference was found in hospital stay between the two groups at the six-month follow-up. Before discharge, hospital stay in the NI and SC groups was 15.4 ± 8.1 days and 15.5 ± 7.4 days, respectively. Six months after discharge, 12 patients in the NI group were readmitted 22 times due to pneumonia (accounting for 22.7%), and 15 patients in the SC group were readmitted 34 times due to pneumonia (accounting for 44.1%). Overall, 17.2% of patients (5/29) in the NI group and 31% of patients (9/29) in the SC group were readmitted due to pneumonia within six months. After adjusting for sex, the readmission rate for pneumonia decreased by 77% in the NI group compared with that in the SC group (*p* = 0.03, OR: 0.228, 95% CI: 0.06–0.87). However, the SC group showed a markedly 1.8-fold higher readmission rate for pneumonia than the NI group, indicating that the iNIP under dietitian support and patient family care reduced readmission due to pneumonia reoccurrence.

## 4. Discussion

To the best of our knowledge, this is the first study to investigate the nutritional outcomes of a prospective iNIP for pneumonia in malnourished older adults. Our results indicated that a six-month iNIP under dietitian support and patient family care for malnourished older adults with pneumonia significantly improved their nutritional status and reduced the readmission rate.

Patients with pneumonia are typically admitted to the hospital. They are prone to reduced calorie intake. A systematic review showed that nearly one-third of hospitalized older adults had decreased physical function, corresponding to a nutrition deficit [32]. Early delivery of enteral nutrition is related to the modulation of stress and the systemic immune response in addition to the reduction of disease severity [33]. In the present study, the results of blood analysis revealed that albumin levels decreased by 0.6 and 0.9 g/dL in the NI and SC groups, respectively. According to the clinical assessment, an albumin level lower than 3.5 mg/dL indicates inadequate nutritional status and significantly affects survival rates [34]. A previous study demonstrated that malnourished older adults with pneumonia receiving gastrointestinal nutrition; significantly increased in serum concentrations of total protein, prealbumin, and retinol-binding protein. These findings suggest the impact of nutrition therapy on patients with pneumonia, especially in older adults [35].

Nutritional support is necessary for patients with inadequate calorie intake, malnutrition, weight loss, and declining respiratory muscle strength [33]. In this study, the calorie intake of the NI group was higher than that of the SC group before discharge and three and six months after discharge (*p* < 0.001, *p* = 0.034, and *p* < 0.001, respectively) (Figure 3). The calorie intake adherence rate was 5.3% higher in the NI group than in the SC group before discharge (*p* = 0.003). Therefore, we postulated that nutritional intervention mechanisms enhanced the nutritional status of the participants as follows. First, nutritional intervention (i.e., NI group) boots immunity and possibly prevents infection. Protein-energy malnutrition and micronutrient deficits disturb immune responses [36]. Older adults who tend to eat less may have a low protein intake, which may lead to loss of muscle mass. In addition to muscle replacement, protein aids in cardiovascular function [37]. Gariballa et al. conducted a randomized, double-blind, placebo-controlled clinical trial to study the effect of nutritional supplementation (i.e., normal hospital diet plus 400 mL oral nutritional supplements) on 445 older adults with acute illness and found that the nutritional status of the intervention group improved, and they were less likely to be readmitted in a six-month follow-up [14]. Snider et al. found that using the Premier Research Database in a sample of 14,326 patients aged ≥65 years with COPD nutritional supplementation was likely to be relevant to decreased numbers of days of hospital stay as well as reduced risk of readmission [15]. Compared with these two other studies, our study focused on malnourished older adults with pneumonia and also on the change in nutritional status and corresponding readmission rate; notably, this nutritional intervention lasted for six months with mutual support from dietitians and family caregivers. Second, the adequate nutritional support may have reduced the oxidative stress in the NI group. Studies have shown that pneumonia may be caused by reactive oxygen species and free radicals formed during infection, which augmented oxidative stress and caused oxidative damage [38]. In particular, the oxidative damage is more evident in malnourished patients than in adequately nourished patients [39,40]. Therefore, the abundance of the essential amino acids in the NI group caused an increase in protein synthesis [41]. However, our study warrants further investigations in the future, including the intake of antioxidant vitamins.

MNA-SF is a nutritional screening tool. Our results showed that MNA-SF scores increased with time in both groups. The MNA-SF scores of the NI group increased by four points on average in six months, and the scores of the SC group increased by 2.4 points on average in six months (Figure 2). Lilamand conducted a one-year follow-up of 773 older residents from nursing homes and used MNA-SF to assess nutritional status; the incidence of pneumonia was 25.6% in patients with a normal nutritional status, 58.7% in those with a risk of malnutrition, and 15.7% in those with malnutrition. After a one-year follow-up, 17.5% of the patients died. Therefore, attention should be paid to the decline in MNA-SF scores, which is an important risk factor; the various scores of MNA-SF should be explored more carefully to verify whether they can be used to detect whether the health status of older adults is declining [42].

The main strength of this study is feasible for enhancing the nutritional intake and nutritional status in malnourished older adults with pneumonia through phone consultation by a clinical dietician for a follow-up time of six months after discharge. An individualized nutritional intervention program was established based on the nutritional needs of a patient identified during admission and was tailored to the individual’s preferences and physical conditions after discharge. The intervention was focused on achieving increased nutritional intake and improved nutritional status, thereby reducing the readmission rate. This limitation of the study was that no blood analysis was performed after discharge. Phone calls are a convenient method for tracking the nutritional intake status, but this method could only assess patients’ calorie intake, intake of the three major nutrients, and MAN-SF scores. No further nutrition-related biochemical data could be obtained.

## 5. Conclusions

A continuous six-month nutritional intervention under mutually dietitian support and family care can improve the caloric intake, protein intake, and MNA-SF scores of malnourished older adults with pneumonia. Although the beneficial effect of this intervention is not obvious during hospitalization, continuous nutritional advice, and follow-up through phone calls can significantly improve nutritional status and reduce the readmission rate.

## Figures and Tables

**Table 1:** Baseline characteristics of the nutrition intervention (NI) and standard care (SC) groups.

Baseline Characteristics	Nutrition Intervention (NI) Group (*n* = 39)	Standard Care (SC) Group (*n* = 43)	*p*
Age (years old)	80.9 ± 7.9	82.2 ± 7.7	0.453
Gender			
male/female	35/4	31/12	0.044 *
Admission diagnosis number of people (%)			0.930
pneumonia, no pathogen	32 (82.0%)	32 (74.4%)	
bacterial pneumonia	2 (5.1%)	4 (9.3%)	
bronchopneumonia	2 (5.1%)	2 (4.6%)	
other pneumonia, no pathogen	3 (7.7%)	4 (9.3%)	
fever caused pneumonia	0 (0.0%)	1 (2.3%)	
Comorbidity Number (%)			
COPD	15 (38.4%)	14 (32.5%)	0.577
DM	13 (33.3%)	14 (32.5%)	0.941
HTN	26 (66.6%)	24 (55.8%)	0.314
CVD	8 (20.5%)	6 (13.9%)	0.430
old stroke	10 (25.6%)	9 (20.9%)	0.614
dementia	11 (28.2%)	3 (6.9%)	0.011 *
other lung diseases	26 (66.6%)	32 (74.4%)	0.001 *

* *p* < 0.05 between groups. COPD, chronic obstructive pulmonary disease; DM, diabetes mellitus; HTN, hypertension; CVD, cardiovascular disease.

**Table 2:** Comparisons of anthropometric measurements between the NI and SC groups.

Anthropometry	NI Group	SC Group	*p* (Interaction)	*p* (Time)	*p* (Group)
BMI (kg/m^2^)			0.345	0.010 *	0.775
T0	20.7 ± 3.4	20.1 ± 4.4			
T1	20.3 ± 3.1	19.8 ± 4.4			
T3	20.3 ± 3.1	20.3 ± 4.4			
T6	20.6 ± 3.1	21.8 ± 5.0			
TSF (mm)			0.767	0.023 *	0.450
T0	15.0 ± 6.8	14.7 ± 6.5			
T1	13.8 ± 6.4	13.6 ± 6.5			
AC (cm)			0.234	<0.001 *	0.417
T0	23.1 ± 2.8	22.4 ± 3.2			
T1	22.6 ± 3.1	22.2 ± 3.4			
AMC (mm)			0.205	<0.001 *	0.745
T0	183.9 ± 29.0	180.8 ± 29.0			
T1	179.8 ± 30.9	178.7 ± 29.3			
AMA (mm^2^)			0.236	<0.001 *	0.709
T0	2757.6 ± 810.1	2667.1 ± 744.3			
T1	2647.4 ± 849.4	2607.9 ± 755.9			

* *p* < 0.05 indicates a significant difference. T0 = during hospitalization; T1 = before discharge; T3 = 3 months after discharge; T6 = 6 months after discharge. BMI, body mass index; TSF, triceps skinfold; AC, mid-upper arm circumference; AMC, arm muscle circumference; AMA, arm muscle area. * *p* <.05. *n* = 39 for NI, 43 for SC at T0; *n* = 39 for NI, 43 for SC at T1; *n* = 31 for NI, 33 for SC at T3; *n* = 29 for both NI and SC at T6, respectively.

**Table 3:** Analysis of blood biochemical values in the NI and SC groups.

Blood Analysis	NI Group	SC Group	*p* (Interaction)	*p* (Time)	*p* (Group)
Albumin (g/dL)			0.953	0.082	0.071
T0	2.7 ± 0.5	1.9 ± 0.4			
T1	2.1 ± 0.3	1.0 ± 0.3			
WBC (µL)			0.054	<0.001 *	0.170
T0	1132.8 ± 5519.6	1391.2 ± 7258.7			
T1	8681.6 ± 3564.6	8556.1 ± 3449.5			
Lym (%)			0.108	<0.001 *	0.885
T0	12.5 ± 6.8	11.2 ± 6.7			
T1	17.4 ± 9.1	18.5 ± 7.9			
TLC (cells/mm^3^)			0.655	0.279	0.606
T0	1284.6 ± 849.8	1432.0 ± 1199.8			
T1	1436.4 ± 924.3	1493.9 ± 803.3			

* *p* < 0.05 indicates a significant difference. T0 = during hospitalization; T1 = before discharge. WBC, white blood cell; Lym, lymphocyte; TLC, total lymphocyte count. n = 39 for NI, 43 for SC at T0; *n* = 39 for NI, 43 for SC at T1; *n* = 31 for NI, 33 for SC at T3; *n* = 29 for both NI and SC at T6, respectively.

**Table 4:** Three-nutrient intake in the NI and SC groups.

Three-Nutrient Intake	NI Group	SC Group	*p* (Interaction)	*p* (Time)	*p* (Group)
Protein (g/day)			0.117	<0.001 *	<0.001 *
T0	52.3 ± 15.2	48.2 ± 14.5			
T1	69.1 ± 10.2	56.5 ± 12.6			
T3	59.7 ± 14.4	51.8 ± 12.6			
T6	62.1 ± 10.5	51.3 ± 11.7			
Lipid (g/day)			0.232	<0.001 *	<0.001 *
T0	48.8 ± 19.0	44.0 ± 12.2			
T1	67.5 ± 23.3	53.7 ± 16.0			
T3	54.8 ± 19.6	44.2 ± 20.5			
T6	57.4 ± 16.7	45.1 ± 15.8			
Carbohydrate (g/day)			0.116	0.811	0.255
T0	183.4 ± 49.9	202.8 ± 111.1			
T1	197.8 ± 46.6	183.2 ± 47.5			
T3	187.3 ± 41.0	187.3 ± 41.0			
T6	203.1 ± 41.2	176.1 ± 40.2			

* *p* < 0.05, indicates a significant difference. T0 = during hospitalization; T1 = before discharge; T3 = 3 months after discharge; T6 = 6 months after discharge. *n* = 39 for NI, 43 for SC at T0; *n* = 39 for NI, 43 for SC at T1; *n* = 31 for NI, 33 for SC at T3; *n* = 29 for both NI and SC at T6, respectively.
